# Structural changes in the cytoplasmic pore of the Kir1.1 channel during pH_i_-gating probed by FRET

**DOI:** 10.1186/1423-0127-16-29

**Published:** 2009-03-06

**Authors:** Jay-Ron Lee, Ru-Chi Shieh

**Affiliations:** 1 Institute of Biomedical Sciences, Academia Sinica, Taipei 11529, Taiwan, Republic of China

## Abstract

Kir1.1 channels are important in maintaining K^+ ^homeostasis in the kidney. Intracellular acidification reversibly closes the Kir1.1 channel and thus decreases K^+ ^secretion. In this study, we used Foster resonance energy transfer (FRET) to determine whether the conformation of the cytoplasmic pore changes in response to intracellular pH (pH_i_)-gating in Kir1.1 channels fused with enhanced cyan fluorescent protein (ECFP) and enhanced yellow fluorescent protein (EYFP) (ECFP-Kir1.1-EYFP). Because the fluorescence intensities of ECFP and EYFP were affected at pH_i _< 7.4 where pH_i_-gating occurs in the ECFP-Kir1.1-EYFP construct, we examined the FRET efficiencies of an ECFP-S219R-EYFP mutant, which is completed closed at pH_i _7.4 and open at pH_i _10.0. FRET efficiency was increased from 25% to 40% when the pH_i _was decreased from 10.0 to 7.4. These results suggest that the conformation of the cytoplasmic pore in the Kir1.1 channel changes in response to pH_i _gating such that the N- and C-termini move apart from each other at pH_i _7.4, when the channel is open.

## Background

K^+ ^homeostasis is controlled by the secretion of K^+ ^ions across the apical membrane of cortical collecting duct cells in the kidney. Low-conductance inwardly rectifying K^+ ^channels are the channels primarily responsible for K^+ ^secretion [1,2]. These low-conductance K^+ ^channels have been shown to be particularly sensitive to changes in the pH_i_. Intracellular acidification in the physiological range reversibly reduces the channel open probability and is thought to account for the subsequent decrease in K^+ ^secretion [1-3]. Thus, the sensitivity of the apical K^+ ^channel to the pH_i _is assumed to play a key role in K^+ ^homeostasis.

The processes involved in the opening and closing of Kir1.1 channels in response to pH_i _changes are not completely understood. It has been suggested that the closure of the Kir1.1 pH_i _gate results from the occlusion of the tetrameric channel pore by the convergence of four leucines at the cytoplasmic apexes of the four inner transmembrane helices [4]. In addition, it was recently proposed that H^+ ^and PIP_2 _use a gating mechanism defined by conformational changes in the transmembrane helices and the selectivity filter and that the gating movement of the transmembrane helices is, in turn, controlled by an intrasubunit hydrogen bond between transmembrane domains 1 and 2 at the helix-bundle crossing [5].

It has been proposed that ligands gating Kir channels open or close the pore by initiating conformational changes in the cytoplasmic domains [5]. The accessibility of N-terminal and C-terminal region cysteines C49 and C308 to methanethiosulfonate reagents has been shown to be pH_i _(state)-dependent, suggesting that pH_i_-gating may involve movements in the cytoplasmic-located pore, which is composed of both the N- and C-terminal regions [6]. Furthermore, the interaction of the N- and C-termini has been suggested to be an important part of the channel gating mechanism [7]. However, there is no direct evidence that the pH_i _can modulate the interaction of the N- and C-termini, either *in vitro *or in intact cells.

In this study, we used patch clamp fluorometry and Foster resonance energy transfer (FRET) microscopy to measure currents and probe the interactions of the N- and C-termini of the Kir1.1 (Kir1.1a) channel during pH_i_-gating. The strength of our approach is that the FRET measurements were performed with simultaneous electrophysiological recordings in inside-out patches with complete control of the intracellular environment. The results showed that the N- and C-termini of the Kir1.1 channel are located closely to each other in the closed (pH_i _7.4) state, and move apart when the channel is opened by a high pH_i_.

## Methods

### Fusion of channels with fluorescent proteins

cDNAs for Kir 1.1 and Kir2.1 channels and the mutant fused with ECFP/EYFP were constructed using commercially available pCMV-ECFP/EYFP vectors (Clontech, Palo Alto, CA). A GGGGGG linker was used to fuse the EYFP to the C terminus of the Kir1.1 and Kir2.1 channels.

### Cell culture

HEK293T cells were cultured in Dulbecco's modified Eagle's medium (Sigma Chemical, St. Lois, MO, USA) containing 10% fetal bovine serum (Life Technologies, Paisley, Scotland) and 1% penicillin-streptomycin at 37°C in a humidified atmosphere containing 5% CO_2_. Cells were plated on poly L-lysine-coated No. 1 glass cover slips (42 mm) (Carl Zeiss, Inc., German) and transiently transfected with 2 μg of plasmids using LipofectAMINE 2000 (Invitrogen Co., Carlsbad, CA, USA) and were used 1–2 days after transfection.

### Preparation of Xenopus oocytes

*Xenopus *oocytes were isolated by partial ovariectomy from frogs anaesthetized with 0.1% tricaine (3-aminobenzoic acid ethyl ester). The fused channel cDNAs were subcloned into the pGEMHE expression vector and cRNAs were obtained by in vitro transcription (mMessage mMachine, Ambion, Dallas, USA). Oocytes were used 1–3 days after cRNA or cDNA injection.

### Preparation of HEK293T cell isolated plasma membrane sheets

Isolated plasma membrane sheets, attached to the cover slip, were prepared by sonication in phosphate-buffered saline (137 mM NaCl, 2.7 mM KCl, 10 mM Na_2_HPO_4_, and 2 mM KH_2_PO_4_, pH 7.4) at 0°C, and incubated at room temperature for 10 minutes before experiments.

### Electrophysiological recordings

Macroscopic currents were recorded at room temperature (21–24°C) using the patch-clamp technique [8,9] and an Axopatch 200B amplifier (Axon Instruments, Foster City, CA, USA). The internal (pH 6 – 10) and external (pH 7.4) 100 mM [K^+^] solution contained 80 mM (KCl + KOH), 5 mM EDTA, and 5 mM HEPES. The command voltage pulses were controlled and data acquired using pClamp6 software (Axon Instruments, Foster City, CA, USA).

### FRET measurement

Fluorescence was acquired using a Zeiss Axiovert 200M microscope (Carl Zeiss, Inc., German) equipped with a mercury lamp and the following filter cubes (nm): (1) CFP cube: EX 436/20, EM 480/40, DCLP 455; (2) EYFP cube: EX 500/20, EM 535/30, DCLP 515; (3) FRET cube: EX 436/20, EM 535/30, DCLP 455 (Omega Optical, Brattleboro, VT, USA). The FRET ratio (FR) was determined using the 3-cube approach [10]. The FR is equal to the fractional increase in EYFP emission due to FRET and is calculated as:

(1) $$\text{FR}=\frac{{\text{F}}_{{\text{A}}_{\text{D}}}}{{\text{F}}_{\text{A}}}=\frac{\left[{\text{S}}_{\text{FRET}}(\text{DA})-{\text{R}}_{\text{D}1}\cdot {\text{S}}_{\text{CFP}}(\text{DA})\right]}{{\text{R}}_{\text{A}1}\cdot [{\text{S}}_{\text{YFP}}(\text{DA})-{\text{R}}_{\text{D}2}\cdot {\text{S}}_{\text{CFP}}(\text{DA})]}$$

In Equation [1] and the definitions of R_D1_, R_D2_, and R_A1_, given below, S_FRET_, S_ECFP_, and S_EYFP _followed by D, A, or DA in parenthesis indicate the fluorescence intensity using the indicated filter cube and cells expressing only the donor, ECFP (D), only the acceptor, EYFP (A), or both (DA). R_D1_, which is equal to S_FRET_(D)/S_ECFP_(D), R_D2_, which is equal to S_EYFP_(D)/S_ECFP_(D), and R_A1_, which is equal to S_FRET_(A)/S_EYFP_(A), are predetermined constants from measurements applied to single cells expressing only ECFP- or EYFP-tagged Kir2.1. R_D1_, R_D2_, and R_A1 _were determined to be 0.51 ± 0.01 (n = 9), 0.0053 ± 0.0001 (n = 9), and 0.26 ± 0.001 (n = 6), respectively. The effective FRET efficiency (E_EFF_) is determined by

(2) E_EFF _= (FR - 1)[ε_YFP_(440)/ε_CFP_(440)]

The bracketed term is the ratio of the YFP and CFP molar extinction coefficients scaled for the FRET cube excitation filter [10] and was previously determined to be 0.094 [10].

## Results and discussion

### A decrease in the pH_i _results in a decrease in ECFP and EYFP fluorescence intensity

**Figure 1:**
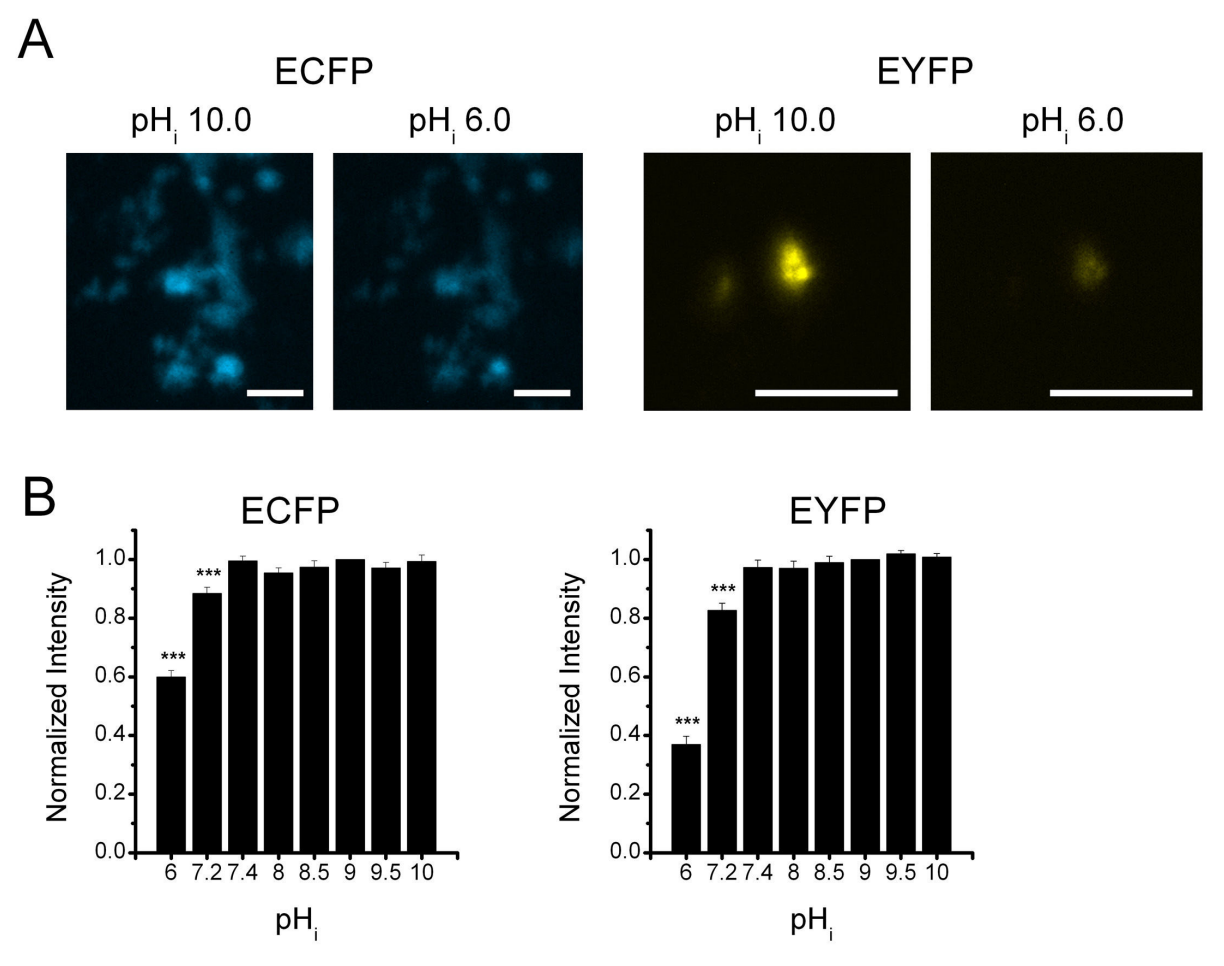
**Effect of the pH_i _on the fluorescence intensities of ECFP or EYFP fused to the Kir2.1 channel**. A. Images of HEK293T cell plasma membrane sheets expressing ECFP-Kir2.1 or Kir2.1-EYFP exposed to pH_i _10.0 or 6.0. For clarity of presentation (but not during data analysis), the contrast of photos was increased by 20% with Photoshop CS3. B. Averaged effect of the pH_i _on the florescence intensities of ECFP-Kir2.1 or Kir2.1-EYFP in plasma sheets (C, n = 15). The fluorescence intensities were normalized to those obtained at pH_i _10.0. *** indicates p < 0.005 compared to the pH_i _10.0 value.

The aim of this study was to examine whether the conformation of the cytoplasmic pore of the Kir1.1 channel changes during pH_i_-gating by measuring the FRET efficiency between ECFP and EYFP fused, respectively, to the cytoplasmic N- or C-terminus of the Kir1.1 channel. However, fluorescent proteins, such as GFP, YFP, and CFP, are pH-sensitive [11,12]. We therefore first determined how the pH_i _affected the fluorescence intensities of ECFP and EYFP expressed near the membrane by fusing them separately to Kir2.1 channels, which are less pH_i_-sensitive than Kir1.1 channels [13]. Figure 1A shows the effects of the pH_i _on Kir2.1-ECFP and Kir2.1-EYFP in HEK293T cell isolated plasma membrane sheets with the cytoplasmic side exposed to the bath. When the bath pH was decreased from pH 10.0 to 6.0, the fluorescence intensities of ECFP and EYFP in isolated membrane sheets decreased (Fig 1A). The "dot structures" shown in Fig 1A are fragmented bottom plasma membranes remain attached to coverslip after sonication of HEK293T cells [5] instead of cytoplasmic organelles. Intracellular organelles did not attached to the membrane after sonication (data not shown) so the pH effects shown in Fig 1A is not due to an acid denaturing artifact caused by intracellular vesicles. Figure 1B shows the averaged effects of various pH_i _values on ECFP and EYFP fluorescence intensities in the isolated membrane sheets (fluorescence intensities normalized to those obtained at pH_i _10.0). The fluorescence intensities of Kir.2.1-ECFP and Kir.2.1-EYFP were stable at pH_i_s ranging from pH 7.4 to 10.0, but a decrease in the pH_i _below 7.4 reduced both in a pH_i_-dependent manner. These results show that the fluorescence intensities of ECFP and EYFP are stable at pHs ranging from 7.4 to 10.0.

### Effects on the pH_i_-gating of fusing ECFP and EYFP to the Kir1.1 channel

**Figure 2:**
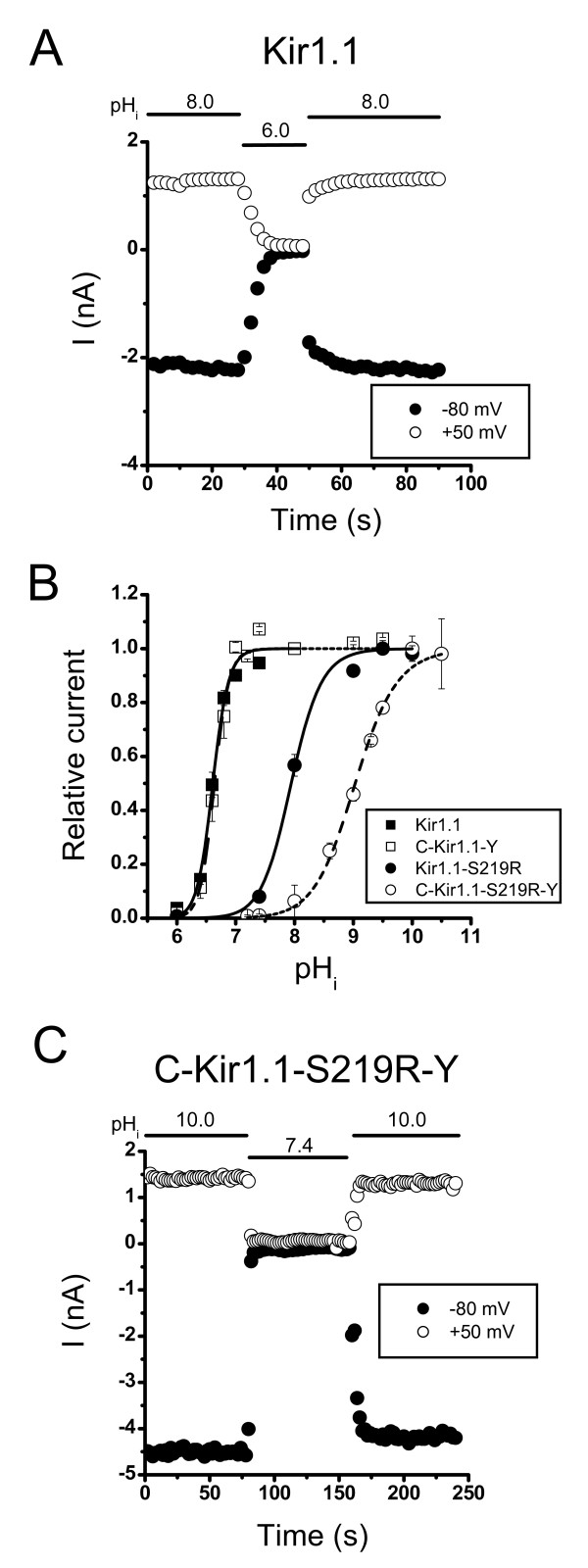
**pH_i_-gating of Kir1.1 channels**. A. Currents were recorded at pH_i _8.0 and 6.0 in an inside-out oocyte patch expressing ECFP-Kir1.1-EYFP. Currents were recorded by holding the membrane potential at -80 mV and stepping to +50 mV for 50 ms every 1 Hz. B. Normalized current-pH_i _relationship. Currents were normalized to those recorded at pH 8.0 for the wild-type Kir1.1 and ECFP-Kir1.1-EYFP construct and to those recorded at pH 10.0 for the S219R mutant and ECFP-S219R-EYFP construct. The smooth curves are the best fit of the data to the Hill equation: 1/{1 + [H^+^]/K_d_]^n^}. n = 3 – 10. C. Currents were recorded at pH_i _10.0 and 7.4 in an inside-out oocyte patch expressing ECFP-S219R-EYFP using the same protocol as in A. C-Kir1.1-Y and C-S219R-Y stand for ECFP-Kir1.1-EYFP and ECFP-S219R-EYFP, respectively.

The Kir1.1 channel is gated in the open state at a pH_i _> 7.4 and a decrease in the pH_i _to 6.0 results in complete closure of the channel [2,3,14]. Consistent with previous studies, the results in Figure 2A show that a decrease in the pH_i _from 8.0 to 6.0 resulted in the complete closure of the ECFP-Kir1.1-EYFP construct at membrane potentials of +50 and -80 mV. Figure 2B shows the pH_i_-dependency of the currents of the unlabelled Kir1.1 channel and that a decrease in the pH_i _reduced channel activities, with a pK_a _of 6.6 and a Hill coefficient of 3.2. To examine whether fusing ECFP and EYFP to the Kir1.1 channel affected channel function, we monitored the effects of pH_i _on the ECFP-Kir1.1-EYFP construct and found that the pH_i_-dependency was the same as that for the wild-type Kir1.1 channel, with a pK_a _of 6.6 and a Hill coefficient of 3.5 (Fig 2B). These results show that fusing ECFP and EYFP to the Kir1.1 channel did not change the pH_i_-sensor or gating. However, in the pH_i _range (6.0 to 8.0) where Kir1.1 channel gating is sensitive to pH_i _changes, the fluorescence intensities of ECFP and EYFP were affected (Fig 1) and the ECFP-Kir1.1-EYFP construct was therefore not suitable for determining whether conformational changes in the cytoplasmic domain are involved in the pH_i_-gating of the Kir1.1 channel using FRET. However, it has been previously shown that the pH_i _gating of the S219R Kir1.1 mutant is shifted to a higher pH_i _range (pHa ≈ 8.0) at which the fluorescence intensities of ECFP and EYFP were stable (Fig 1). We therefore examined the effect of the pH_i _on channel gating in the S219R mutant and the ECFP-S219R-EYFP construct. Consistent with previous results [14], the pH_i_-dependency curves for S219R and ECFP-S219R-EYFP were shifted to higher pH_i _values compared to that for the wild-type Kir1.1 channel. The S219R and ECFP-S219R-EYFP mutants were completely closed at pH_i _7.4, but fully open at pH_i _10.0 (Figs 2B and 2C). For the S219R mutant, the pK_a _was 8.0 and the Hill coefficient 1.4, while the corresponding values for the ECFP-S219R-EYFP construct were 9.0 and 1.2. These results suggest that the fusion of ECFP and EYFP to the S219R mutant affects the structure near the pH_i _sensor, but does not disrupt gating in response to pH_i _changes. In summary, the ECFP-S219R-EYFP construct was suitable for studying whether the FRET efficiency was affected by pH_i_-gating.

### The FRET efficiency of the ECFP-S219R-EYFP construct is reduced when the channel is in the open state

**Figure 3:**
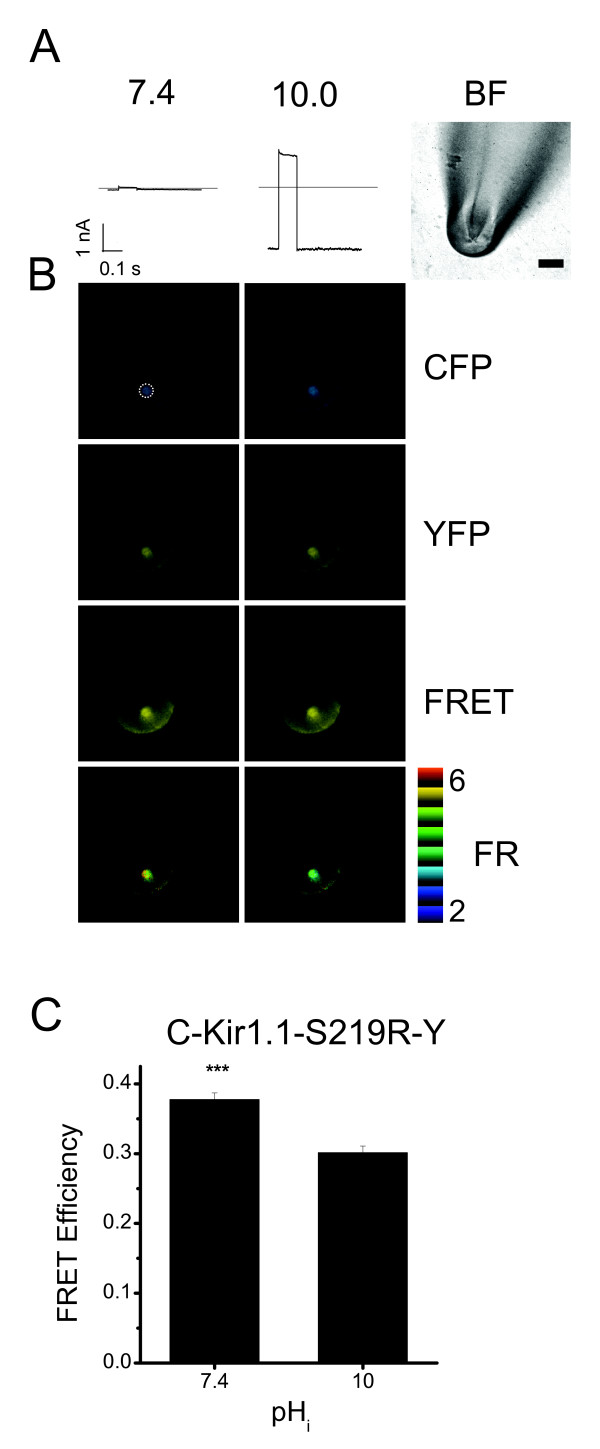
**Effects of the pH_i _on the FRET efficiency**. A. Bright field image of a pipette containing a oocyte membrane patch (right panel) and currents recorded at +50 mV from a holding potential of -80 mV at pHi 7.4 (left panel) or pHi 10.0 (center panel). B. Fluorescence images acquired with the ECFP, EYFP, and FRET filter cubes at the indicated pH_i_. Fluorescence intensities were measured in the area of the membrane patch indicated by the dotted circle. Scale bar, 20 μm. C. Averaged FRET efficiency of ECFP-S219R-EYFP in the open state (pH_i _10.0, n = 6) and closed state (7.4, n = 8) and FRET efficiency of ECFP + EYFP. n = 6 – 8. *** indicates p < 0.005 compared to the pH_i _10.0 value.

Figure 3 shows the simultaneous recordings of fluorescence intensities and currents in an inside-out oocyte patch expressing ECFP-S219R-EYFP. The right panel of Figure 3A shows the bright-field image of a pipette tip containing a membrane patch, while the other panels show current recordings at pH_i _7.4 and 10.0. The same membrane patch showed strong fluorescence recorded with CFP, YFP, and FRET cubes at pH_i _7.4 and 10.0 (Fig 3B). The FRET ratio calculated using the 3-cubes method is shown in pseudo-colors. As reported previously [15], the periphery of the pipette tip was also fluorescent due to membrane stuck to the glass, but the thickness of the pipette provided a good separation of the fluorescence signals from the clamped membrane patch and the membrane stuck to the glass. Figure 3C shows the averaged FRET efficiency at pH_i _7.4 and 10.0 for the ECFP-S219R-EYFP construct. The FRET efficiency of the construct was large, indicating that the N- and C-termini are close to each other at pH_i _7.4 (closed state), and was significantly decreased when the pH_i _was increased from 7.4 to 10.0. To confirm our findings, we coexpressed cytoplasmic ECFP and EYFP in HEK293T cells. Figuure 3C shows that in cells co-expressing ECFP and EYFP, the FRET ratio was 1.14 ± 0.04 and the FRET efficiency 1.3 ± 0.1%, indicating no interaction between free ECFP and EYFP. The control experiment supports that the 3-cube FRET method can effectively avoid the potential leaking signal problem, consistent with several previous studies [10,16-18]. These results show that the FRET efficiency of ECFP-S219R-EYFP is smaller when the channels are in the open state than in the closed state and suggest that the N- and C-termini move apart from each other in response to pH_i_-gating in the mutant. Previous study [14] and our Fig 2B show that the S219R mutant is still able to open and close in response to pH_i _changes. The results suggest that it is probably the pH_i _sensor that is changed in the mutant and the conformation processes associated with pH_i _changes are intact in the S219R mutant. Therefore, it is likely that the relative movements of N- and C-termini are similar in both the wild-type Kir1.1 and S219R channels. However, we cannot rule out that the dynamic changes of channel conformations in the S219R mutant are different from those in the wild-type Kir1.1 channel. Furthermore, the conjugation of ECFP and EYFP to the S219R but not the Kir1.1 channel affects its sensitivity to pH_i _changes, suggesting that ECFP and EYFP may affect pH_i _sensing and/or the gating of channel in response to pH_i _changes. Further investigations using different fluorescence probes or different techniques are required to understand the conformational changes of the wild-type Kir1.1 channel.

Based on FRET measurement, it has been suggested that the opening of the Kir3.x channel upon G-protein stimulation induces a rotation of the C-terminal and a decrease in the distance between the N- and C-termini of adjacent subunits [17]. In contrast, we showed that the distance between the N- and C-termini increased when the Kir1.1 channel was open (high pH_i_). These results support the hypothesis that ligands gating Kir channels initiate conformational changes in the cytoplasmic domains that are transduced to the transmembrane helices and thereby open or close the pore [5]. However, the details of the conformational changes in the cytoplasmic pore in response to ligand gating may vary in different Kir channels. Further experiments are required to understand how the movements of the cytoplasmic pore are transduced to the transmembrane helices.

## Competing interests

The authors declare that they have no competing interests.

## Authors' contributions

JRL conducted the experiments and analyzed the data whereas RCS designed experiments and wrote the manuscript.
